# Transcriptome Analysis Reveals Functional Diversity in Salivary Glands of Plant Virus Vector, *Graminella nigrifrons*

**DOI:** 10.3390/genes11111289

**Published:** 2020-10-29

**Authors:** Swapna Priya Rajarapu, Raman Bansal, Priyanka Mittapelly, Andrew Michel

**Affiliations:** 1 Department of Entomology and Plant Pathology, North Carolina State University, Raleigh, NA 27606, USA; 2 Department of Entomology, The Center for Applied Plant Sciences, OARDC, The Ohio State University, Wooster, OH 44691, USA; raman.bansal@usda.gov (R.B.); priyanka.mittapelly@gmail.com (P.M.); michel.70@osu.edu (A.M.); 3 USDA-ARS, San Joaquin Valley Agricultural Sciences Center, 9611 South Riverbend Avenue, Parlier, CA 93648, USA; 4 USDA-APHIS PPQ, 5936 Ford Court, Suite 200, Brighton, MI 48116, USA

**Keywords:** salivary gland, transcriptome, planthopper

## Abstract

Insect salivary glands play an important role for host feeding, specifically by secreting salivary proteins for digestion and potentially modulating host defenses. Compared to other hemipterans, the significance of salivary glands is less studied in the black-faced leafhopper, *Graminella nigrifrons*, a crop pest that vectors several agronomically important plant viruses. To identify functionally important genes in the salivary glands of the black-faced leafhopper, we compared transcriptomes between adult salivary glands (SG) and the remaining carcasses. We identified 14,297 salivary gland-enriched transcripts and 195 predicted secretory peptides (i.e., with a signal peptide and extracellular localization characteristics). Overall, the SG transcriptome included functions such as ‘oxidoreduction’, ‘membrane transport’, and ‘ATP-binding’, which might be important for the fundamental physiology of this tissue. We further evaluated transcripts with potential contributions in host feeding using RT-qPCR. Two SG-enriched transcripts (log_2_ fold change > 5), *GnP19* and *GnE63* (a putative calcium binding protein), were significantly upregulated in maize-fed adults relative to starved adults, validating their importance in feeding. The SG-enriched transcripts of the black-faced leafhopper could play a potential role for interacting with maize and could be targets of interest for further functional studies and improve pest control and disease transmission.

## 1. Introduction

The interactions between plants and insects has long been a model for understanding adaptive evolution as well as explaining the emergence of insect crop pests [1,2]. Plant defense against herbivory plays a crucial role in this interaction, exerting selection pressure on insects. Correspondingly, insects evolved molecular and physiological adaptations, such as detoxification, target site modification, and sequestration of the defensive chemicals, to overcome plant defenses after feeding [3]. Some of these adaptations occur prior to or during feeding which alter or manipulate plant defenses [4,5]. Such mechanisms are mediated by the salivary glands, producing saliva that is released into the plant to facilitate feeding. Often, these salivary proteins lead to novel and specific plant–insect interactions.

Most of the existing knowledge on insect salivary proteins come from vascular feeders with piercing, sucking mouthparts in the order Hemiptera. This order contains two main suborders, the Sternorrhyncha (which includes aphids, whiteflies, plant lice, and scale insects) and the suborder Auchenorrhyncha (cicadas, froghoppers, treehoppers, planthoppers, and leafhoppers). Both suborders secrete non-soluble, gel saliva and soluble watery saliva to promote plant feeding. Proteins present in the gel saliva help form and maintain the salivary sheath and potentially interact with the plant. Alternatively, components in the watery saliva play a larger role in digestion as well as for interacting with plant defense mechanisms. For example, plants can defend against vascular feeders by using calcium to block the sieve elements from feeding and/or by eliciting an oxidative burst response. Calcium-binding proteins in the watery saliva can then prevent blockage or suppress a reactive oxygen burst [6,7,8].

Many biological and behavioral differences exist among the suborders Sternorrhyncha and Auchenorrhyncha, including their interactions and feeding mechanisms with host plants. Sternorrhyncha feed intracellularly, moving mouth parts between cells to reach the phloem, whereas Auchenorrhyncha pierce plant cells for feeding [9]. These differences in plant feeding may be related to the differences in specific salivary responses shown by transcriptomic studies. For example, aphids and whiteflies have less than 10 salivary transcripts encoding for the common detoxification proteins cytochrome P450 and glutathione-S-transferases, whereas planthoppers contain as many as 4–5 times the number of these transcripts [10,11]. While much is known regarding the salivary transcriptomes of members of Sternorrhyncha, resources for Auchenorrhyncha are limited.

The black-faced leafhopper, *Graminella nigrifrons*, is one of the most commonly found leafhoppers, with a wide distribution in the United States [12]. It has a wide host range on many grass species including grain crops, such as maize and sorghum [13]. It also vectors debilitating plant diseases such as corn stunt virus, maize chlorotic dwarf virus, maize fine streak virus, and phytoplasmas [14,15,16]. Its ability to vector multiple pathogens and feed on a wide range of hosts makes it a significant agricultural pest, yet little is known about the molecular interactions between the black-faced leafhopper and its host plants. Identifying the molecular components of black-faced leafhopper saliva will expand our understanding of plant feeding in this insect vector and among other related hemipteran species.

Using transcriptomics, we characterized the molecular expression of salivary glands from the black-faced leafhopper and identified salivary gland transcripts that were both enriched and predicted to be secreted in the saliva. We hypothesized that SG-enriched transcripts with signal peptides are expressed upon feeding on host plants relative to non-feeding status. We developed two transcriptomes: one from dissected salivary glands and the other from the remaining carcasses of maize-fed black-faced leafhopper adults. Transcriptomes were assembled and annotated to identify putative secretory peptides with increased expression in the salivary gland relative to the carcass. We then chose five SG-enriched candidate transcripts (log_2_ fold change > 5) to test the hypothesis of increased gene expression in maize-fed adults compared to starved adults. Our study represents the first salivary gland transcriptome of the black-faced leafhopper and identifies important salivary gland transcripts relevant for feeding.

## 2. Experimental Procedures

### 2.1. Insects

We established a laboratory colony of the black-faced leafhopper from collections in maize fields around the Ohio Agricultural Research and Development Center campus (40.773 N–81.909 W) [17]. The black-faced leafhopper colony was fed either 2–3 week-old maize or oats at 24 °C and maintained at 40% relative humidity and 14 L: 10 D photoperiod. Plants were replaced weekly and watered when required. Fully mature adults (identified by wing development) were collected by an aspirator off the plants for dissections.

### 2.2. Total RNA Isolation and Library Preparation

Salivary glands from 40 black-faced leafhopper adults (7–10 days old) were dissected in 1X phosphate-buffered saline (pH 7.0) under a dissecting microscope. The salivary glands and carcass (i.e., whole body minus salivary glands) from 40 black-faced leafhopper adults were pooled, constituting one biological replicate (three replications were included for each treatment). Samples were processed for total RNA extraction using the PureLink^®^ RNA Mini Kit (Life Technologies Corporation, Carlsbad, CA, USA) following the manufacturer’s protocol. To remove DNA contamination, samples were treated with PureLink^®^ DNase (Life Technologies Corporation, Carlsbad, CA, USA). RNA quality was checked using a Nanodrop 2000c (Thermo Scientific, Hudson, NH, USA) and an Agilent Bioanalyzer 2100 (Agilent Technologies, Palo Alto, CA, USA). The cDNA libraries for RNA-Seq were prepared using the TruSeq RNA Sample Preparation Kit (Illumina Inc., San Diego, CA, USA) following the manufacturer’s protocol. Briefly, 4 μg of total RNA from each sample was used to purify and fragment mRNA (library insert fragmentation at 94 °C for 8 min to give an insert of 155 bp; range 120–210 bp), followed by first and second strand cDNA synthesis. We followed a series of steps including end-repair (to convert the overhangs resulting from fragmentation into blunt ends), adenylation of 3′ ends of the blunt fragments (to prevent them from ligating to one another during the adapter ligation reaction), ligation of adapters to the ends of double stranded cDNA, and PCR amplification to enrich DNA fragments with adapters. Unique adapter sequences were included for each of the three biological replicates from each treatment. The high quality of the libraries was confirmed using a high sensitivity DNA chip on Agilent Bioanalyzer 2100 (Agilent Technologies, Palo Alto, CA, USA). The libraries for the three biological replicates of each treatment (salivary glands and carcasses) were pooled, and the pooled sample was sequenced in different lanes of a HiSeq 2000 flow cell (Illumina Inc., San Diego, CA, USA). The paired-end sequencing (read length of 100 nucleotides) was performed at the Core Facility, The Ohio State University, Columbus, OH, and all data was deposited in National Center for Biotechnology Information under accession number PRJNA495693.

### 2.3. Transcriptome Assembly and Differential Expression Analysis

We assessed the quality of the 150 bp paired end reads using FastQC (v.0.10.1) [18]. Libraries were also screened for the presence of any rRNA and adapter contamination using the FastQ screen (v.0.11.1) [19]. Along with adapters and rRNA contaminants, we removed low-quality reads (those with a read length lower than 40 bp and a quality score <32) using BBduk (v.36.64) in BBmaptools package [20]. A reference transcriptome for generating count data was assembled de novo using Trinity (v.2.2.0) [21] with default parameters. The quality of the assembled transcriptome was determined by TransRate (v.1.0.3) [22] and Benchmarking Universal Single-Copy Orthologs (BUSCO) (v.3.1.0) against the invertebrate database [23]. Redundant transcripts with 99 % similarity were removed using CD-HIT (v.4.6.1) [24] to create a non-redundant reference transcriptome for differential expression analysis. Count data for individual treatments was generated using RSEM (v.1.2.16) [25], and differentially expressed genes were identified using DESeq2 (v.1.15.46) [26]. For this study, we considered transcripts with P_adj_ < 0.05 and a log_2_ fold change ≥5 as enriched in salivary gland. Differentially expressed transcripts were annotated against NCBI non-redundant database (downloaded on 23 April 2020) with BLASTx and an e-value cut-off of 10^−5^. Enriched gene ontology terms in up- and down- regulated transcripts were identified against the total differentially expressed proteins by one-sided Fisher’s exact test with a False discovery Rate (FDR) threshold of 0.05 in Blast2GO software.

### 2.4. Functional Annotation of the Salivary Gland Transcriptome

To identify transcripts encoding proteins released into the plant, we functionally characterized the salivary gland transcriptome using Trinotate (v.3.0.1) [27]. Trinotate uses TransDecoder to predict protein sequences in silico, which are then blasted against the non-redundant Uniprot database. The predicted proteins were further analyzed in Blast2GO [28] to identify the qualitative distribution of gene ontology terms within this tissue. Putative secretory peptides were identified using SignalP (v.5) [29], and the presence of a transmembrane domain was identified using the TMHMM server (v.3.1) [30]. Localization of translated proteins with a signal peptide and the absence of transmembrane domain was predicted by DeepLoc (v.1.0) [31]. Gene ontology term distributions within the putative secretory proteins were identified in Blast2GO. Additionally, unannotated proteins within the secretory peptides were annotated by InterProScan [32].

### 2.5. Real Time Quantitative PCR (RT-qPCR) for Expression Analysis in Maize-Fed vs. Starved Insects

We used RT-qPCR to quantify the levels of candidate transcripts in the heads and carcasses of maize-fed and starved black-faced leafhopper adults to test our hypothesis that SG-enriched transcripts encoding secretory proteins respond to feeding on host relative to non-feeding or starving status. As an artificial diet has not yet been developed for the black-faced leafhopper, our starved treatment served as our control. Although starved insects may not provide the same transcriptional response as an artificial diet, they provide a control to evaluate responses to general feeding. In a Petri dish, we isolated 50 adults each from three colonies (reared on oats) and provided only moist filter paper for 24 h to clear the gut contents. After this initial starvation period, we separated the adults into two cohorts: one was fed maize and the other was starved for an additional 24 h (48 h total). For the fed cohort, we placed 20 adults on two-week-old maize plants, potted in four-inch pots, and allowed them to feed for 24 h. A transparent tube cage covered the maize plant to allow the insects to move and feed freely. For the starved treatment, 20 adults were placed in an empty Petri dish with moist filter paper as explained above. Adults from the treatments were collected and maintained on ice until decapitation. We observed insignificant mortality in the starved treatment (data not shown) and only the surviving insects were collected for RNA isolations. We collected 12 adults, on average, per each replicate for the starved treatment and 15 adults per each replicate in the fed treatment. For this experiment, heads with salivary glands and carcasses of the insects from both the treatments were collected in Trizol (Invitrogen, Waltham, MA, USA) for RNA isolation. Extraction of total RNA with Trizol resulted in better yields from smaller amounts of tissue.

Total RNA was isolated according to the manufacturer’s protocol, and quality was assessed using a Nanodrop8000 spectrophotometer (Thermo Scientific, Waltham, MA, USA). Total RNA was normalized to the sample with the least amount of RNA and used for cDNA synthesis with the iScript™cDNA synthesis kit (Bio-Rad, Hercules, CA, USA). Primers for the candidate genes were designed using the Primer3 web-based tool. Primer efficiencies were calculated from the slope of the standard curve, developed from a serially diluted cDNA template (Supplementary File 1: Table S4). The expression level of the candidate genes was quantified using a Bio-Rad thermocycler (Hercules, California, USA) with the following parameters: 95 °C for 3 min followed by 40 cycles of 95 °C for 30 s and 55–60 °C for 15 s. To determine the specificity of the primers, PCR products were analyzed on an agarose gel, and melt curves were analyzed on the Bio-Rad thermocycler. The black-faced leafhopper-specific housekeeping gene *GnRPS13* was used as a reference gene to normalize the expression of the target genes in this study [33]. Relative transcript abundance values obtained by normalizing the transcript levels of the transcript of interest to *GnRPS13* were used for statistical tests. Differences in the mean transcript levels of candidate genes tested in the head and carcass of starved and fed black-faced leafhopper adults were compared by two-way ANOVA in Minitab 17 to compare the mean expression levels of the candidate transcripts between the treatments and tissues. The main effects, treatment, tissues, and the interactions were analyzed using an α of 0.05. Tukey’s multiple testing correction was used for comparison of significant main levels. Residual plots were assessed to determine the model fit. Relative transcript levels of all genes had a normal distribution with homogenous variance.

## 3. Results

### 3.1. Deep Sequencing and Assembly of the Salivary Gland and Carcass Transcriptomes 

We obtained >40 million paired end reads from the salivary glands and >58 million paired end reads from the carcass. After quality assessment (curation of raw reads, including trimming of adapters and removal of low-quality reads), our final sequencing set retained 85–88 % of the raw reads, totaling ~35 million and ~45 million paired end reads from the salivary glands and the carcass, respectively (Supplementary File 1: Table S1). All libraries had <1 % rRNA contamination. We assembled two transcriptomes: a salivary gland transcriptome (69,641 transcripts) to identify putative secretory peptides and a reference transcriptome (136,865 transcripts) to identify SG enriched transcripts (Table 1). Optimal TransRate score for the assembled transcriptome was 0.195, and BUSCO analysis showed 80 % complete BUSCOs and 65.6 % complete and single copy BUSCOs (Supplementary File 1: Table S2), which is similar to other arthropods and vascular feeders [22,34,35].

### 3.2. Characterization of the Salivary Gland Transcriptome and its Predicted Proteome

**Figure 1:**
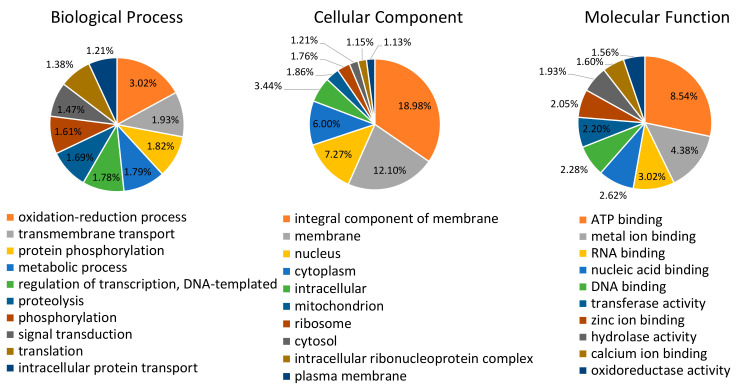
Top ten level two gene ontology distribution in ‘biological process’, ‘cellular component’, and ‘molecular function’ in the salivary gland transcriptome of *Graminella nigrifrons*, black-faced leafhopper, as determined by Blast2GO.

*In silico* translation of the salivary gland transcriptome resulted in 13,842 predicted peptides, of which 9145 had annotations. We further characterized the annotated peptides using Blast2GO to determine the gene ontology (GO) distribution within the transcriptome and identify the putative secretory proteins in the saliva. To determine the functional distribution within the salivary gland transcriptome, we focused on the top ten represented GO terms (level two) within the categories of ‘biological process’, ‘molecular function’, and ‘cellular component’ (Figure 1). Peptides with ‘oxidoreduction’, ‘ATP-binding activity’, and ‘integral component of membrane’ and ‘membrane’ associated transcripts were higher in the biological process, molecular function, and cellular component GO terms, respectively.

Within the ‘cellular component’ category, we filtered for peptides that were likely localized extracellularly and found a total of 49 annotated peptides (Supplementary File 1: Table S3): 25 in the extracellular region and 24 in the extracellular space. Predicted peptides within the extracellular region included chitin deacetylases, chitinases, peptidases, lipases, and carboxylesterases. Predicted peptides localized in the extracellular space included peptidases, superoxide dismutase (SOD), hydrolases, and isomerase. In addition, we observed 46 predicted peptides of cytochrome P450s and 16 glutathione-S-transferases, which are well-studied detoxification enzymes in herbivorous insects.

Typical secretory or salivary proteins are identified in silico with presence of a signal peptide and absence of transmembrane domain, as these are released outside the cell [36,37]. In addition to these features, we included proteins with extracellular localization as potentially secreted into the saliva. With these qualifying characteristics, we found 195 predicted peptides, 115 annotated and 80 uncharacterized peptides (Supplementary File 2: Table S5). Molecular function gene ontology (level four) categorized the peptides into ‘ester bond hydrolase activity’, ‘glycosyl bond hydrolase activity’, ‘chitin-binding’, ‘peptidase activity’, and ‘cation-binding’ (Table 2). Predicted peptides within these categories included proteins predominantly identified in the salivary proteins of other hemipterans such as cathepsin B, cellulose-binding, and lipases. In addition, an InterProScan of the unannotated proteins annotated only 3.75 % of the proteins, which indicates the specificity of these proteins to the black-faced leafhopper.

### 3.3. Differential Expression of Transcripts Between the Salivary Glands and Carcass

Comparing transcriptome profiles of salivary glands and the carcass provided evidence for SG-enriched transcripts. In total, we found 14,297 differentially expressed transcripts (P_adj_ < 0.05) and 5998 transcripts with a fold change of more than five. More than half of the upregulated salivary gland transcripts had log_2_ fold change >5 (3524; 58.7%) (Supplementary File 2: Table S6). Among the differentially expressed transcripts with log_2_ fold change >5, only 26.6% were annotated, whereas 70.1% were not annotated, 1.5% were uncharacterized proteins, and 1.8% were hypothetical proteins. Enrichment analysis of the upregulated transcripts (FDR = 0.05) identified ‘ester hydrolase activity’ (122 transcripts, FDR = 0.028) and ‘extracellular region’ (95 transcripts, FDR = 0.042) gene ontologies enriched in molecular function and cellular component categories, respectively.

Among the upregulated transcripts, the salivary glands contained transcripts for antioxidant and detoxification enzymes, such as laccase and esterase, respectively (Table 3). Among transcripts that are known to have effector-like features in other hemipterans, the salivary glands of the black-faced leafhopper have a higher expression of six calcium-binding transcripts and one cell wall-degrading enzyme (Table 3). Notably, we found higher expression of transcripts homologous to four proteins secreted in the saliva of the green rice leafhopper (*Nephotettix cincticeps*): NcSP19, NcSP75, NcSP22, and NcSP84 [38]. The homolog of NcSP19, named *GnP19* in this study, had a log_2_ fold change of 12.29 in the salivary glands relative to carcass and shared 63.6 % sequence similarity with NcSP19. The homolog of NcSP75, named *GnP75*, had a log_2_ fold change of 9.31 in the salivary glands relative to carcass and shared 43 % similarity with NcSP75. Homologs NcSP22 and NcSP84 shared 48.1 % and 59.2 % similarity with a fold change of ten.

**Figure 2:**
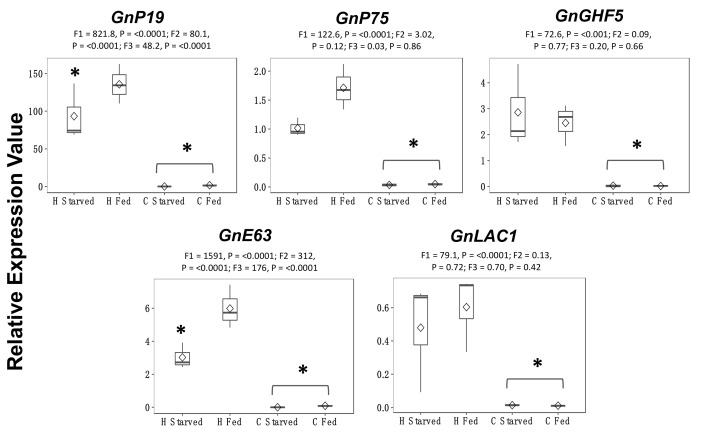
Relative expression values of candidate genes (log_2_ > 5) in the heads (H) and carcasses (C) of black-faced leafhopper (*Graminella nigrifrons*) adults fed on maize relative to starved adults. Transcript levels were normalized to an endogenous reference gene (*GnRPS13*) and fold changes were calculated relative to transcript levels in starved insect tissues. Each bar represents mean ± *SE* from three biological replicates. Differences in mean are calculated by two-way ANOVA with α = 0.05 and Tukey’s multiple correction test for main effects. F1: F statistic for tissues; F2: F statistic for treatment; F3: S statistic of the interaction terms. Candidate transcripts: *GnP19* (unannotated), *GnP75* (unannotated), *GnGHF5* (GHF5 cellulases), *GnE63* (Calcium binding protein), and *GnLac1* (Laccase1). Statistical difference between the tissues is shown with an asterisk over the brackets, and significant difference between the treatments within the tissue is denoted by the asterisk over the bars.

To understand the role of SG-enriched transcripts in the black-faced leafhopper feeding, we quantified the expression level of a few selected transcripts in maize-fed and starved adult heads using RT-qPCR. The selected candidates had functional evidence available and were also identified in the saliva of other hemipterans, such as candidate transcripts homologous to the green rice leafhopper saliva (*GnP19* and *GnP75*) [38] and transcripts encoding antioxidant enzymes (laccase, *GnLac1*) [39], cell wall degrading (endoglucanase, *GnGHF5*) [40], and calcium binding protein (*GnE63*) [41]. We predicted that highly expressed transcripts in maize-fed adults are essential during feeding and would be important for black-faced leafhopper–host interactions. Among the transcripts studied, only *GnE63* and *GnP19* had significantly higher expression in maize-fed adults than starved adults (Figure 2). However, we observed a statistical interaction between the two treatments, tissue and starved vs fed treatment, indicating the responses of these two transcripts is also tissue dependent.

## 4. Discussion

Characterizing the transcriptome of salivary glands is the first and essential step to understanding the intricate relationship between insect vector of plant viruses and their host plants. For black-faced leafhopper, we profiled the salivary gland transcriptome, identified putative secretory peptides, and validated five SG-enriched transcripts associated with feeding on maize. The salivary gland transcriptome encompassed a repertoire of transcripts encoding proteins vital for digestion as well as potentially interfering with plant defense responses. Moreover, the transcripts shared similarity with salivary transcripts and proteins from other related species, such as the green rice leafhopper [42]. Overall, our study highlighted salivary gland transcripts that potentially facilitate black-faced leafhopper–plant interactions.

Similar to other hemipterans, the salivary gland transcriptome represented vital biological functions important for tissue maintenance and host feeding. Peptides with an ‘oxidoreduction’ function were higher in the biological process category relative to the other processes. A majority of peptides in the oxidation–reduction category were housekeeping genes and might participate in maintaining salivary gland functions. Cells of phytophagous insect salivary glands are enriched with endoplasmic reticulum [43], an organelle that maintains the oxidation–reduction environment of the cell and also supports the secretory activity of this tissue. Although functional evidence is needed, our data implicates the importance of oxidoreduction activity in salivary glands [44]. Within the ‘molecular function’ category, peptides with ‘ATP-binding activity’ were abundant. The secretion of molecules into the saliva requires the establishment of a proton gradient that is facilitated by the V-ATPase pump in the apical plasma membrane [45]. These pumps require ATP to actively transport molecules across the tissue, which could explain the high levels of ATP-binding activity in the salivary glands. The ‘cellular component’ GO term contained abundant predicted peptides in the categories of ‘integral component of membrane’ and ‘membrane-associated transcripts’, further emphasizing the function and importance of membrane transporters in the salivary gland tissue for moving molecules across the lipid bilayer.

Proteins or molecules secreted in the saliva typically have extracellular localization, either in the extracellular region or extracellular space. Proteins in the extracellular region remain associated with the cell (i.e., membrane proteins that are externally bound to the lipid bilayer), whereas the extracellular space proteins are secreted out of the cell. Enrichment of transcripts encoding proteins with ‘extracellular region’ gene ontology term supports the secretory role of salivary gland tissue, which is essential for host feeding in piercing-sucking insects. The number of predicted secretory proteins are comparable to those found in the saliva of the brown planthopper, *Nilaparvata lugens* [10]. Most of these proteins are enzymes that could either participate in the fundamental physiology of the salivary gland tissue or host utilization.

Carbohydrate-degrading enzymes, such as chitin deacetylases and chitinases, play a role in cuticle reorganization [46] and are identified within the ‘extracellular region’ GO term in black-faced leafhopper transcriptome. Salivary glands are lined with a cuticle, possibly to maintain a separation between the salivary duct lumen contents and the hemolymph in order to prevent autointoxication [43,45]. We hypothesize that chitin deacetylases and chitinases might help to reorganize the salivary duct cuticle after each feeding cycle. Alternatively, these enzymes could also play a role in breaking down plant cell walls. Members of Auchenorrhyncha likely release cell wall-degrading enzymes to help pierce through the cell wall and extract the vascular contents [9]. Cell wall-degrading enzymes, such as cellulase family glycosyl hydrolase (*GnGHF5*), were expressed nine-fold higher in the salivary glands relative to carcass. A similar cell wall-degrading enzyme, endo-1,4-β-glucanase, is also present in the watery saliva of the green rice leafhopper and was hypothesized to degrade the cell wall of the host [38].

Lipases and carboxylesterases are the abundant hydrolases represented in the hydrolase enzyme class within the salivary gland tissue of black-faced leafhopper. Lipases are primarily digestive enzymes present in other hemipteran salivary gland transcriptomes [42,47]. These enzymes likely degrade the cell wall or digest host proteins, whereas carboxylesterases might be involved in hydrolyzing endogenous toxins [48,49]. Ester hydrolase activity was also enriched within the upregulated transcripts in the salivary gland tissue and might be essential for interacting with the plant host or as a part of the constitutive metabolism of the tissue. Salivary glands of other planthoppers and leafhoppers also contain esterase [10,42], where these hydrolytic enzymes are proposed to detoxify plant secondary metabolites [50]. We predict similar detoxification functions of esterase in black-faced leafhopper salivary glands.

Enzymes such as SOD are important to neutralize reactive oxygen species, particularly superoxide ions encountered from both endogenous and exogenous sources [48]. Plants release reactive oxygen ions in the form of oxidative bursts at feeding sites [6]. By releasing SOD while feeding, an insect can inhibit this plant defense response. SOD has been identified in the watery saliva of the green rice leafhopper [38]. In addition to SOD, a glutathione peroxidase was also identified in the SG-enriched transcripts, which is also an antioxidant enzyme that neutralizes toxic peroxide ions. 

The transcriptome included cytochrome P450 and glutathione transferases, but their role in salivary glands is probably limited to the detoxification of endogenous toxins. Rapid penetration of plant membrane during feeding might result in the uptake of the plant secondary chemicals [51]. By expressing a suite of detoxification enzymes in salivary glands might negate the effect of these chemicals. These findings also correlate with the other hemipteran salivary gland profiles, emphasizing the highly conserved functions across the different members [10,47,52,53,54].

Calcium-binding proteins have been functionally determined to interact with plant defenses and are broadly present in the saliva of hemipterans as well as within the SG-enriched transcripts of black-faced leafhopper. Calcium-binding proteins prevent plant-based calcium from forming sieve plugs and signaling other early defense responses in plants [41,55]. In our study, *GnE63* transcript responded to feeding on maize. Since *GnE63* has signatures of a calcium-binding protein, we predict that the black-faced leafhopper may use it to suppress the early signaling of plant defenses.

In addition to the typical salivary gland enzymes, we also identified transcripts homologous to the salivary proteins of green rice leafhopper. NcSP84 was identified as a potential calcium-binding protein, whereas the function of NcSP22 was not determined. In the green rice leafhopper, NcSP75 is expressed only in the salivary glands and has no homology to other insects, whereas NcSP19 helps form the salivary sheath [38]. Given their high similarity and presence of signal peptide for *GnP19* and enrichment in salivary glands, we hypothesize that *GnP19*, *GnP75*, and the homologs of NcSP84 and NcSP22 might play an important role in interactions of black-faced leafhopper with maize. *GnP19* had 1.6-fold higher expression in maize-fed heads relative to the starved controls and also higher expression in heads relative to the carcass tissue, thus corroborating our RNA-seq findings (Figure 2). Functional characterization using RNA interference will help in understanding the importance of these three transcripts in leafhopper–host interactions.

Our research shows that the salivary gland transcriptome of black-faced leafhopper exhibits molecular and physiological functions similar to other well-characterized Auchenorrhyncha salivary glands. These transcriptomes collectively highlight the importance of membrane transporters to the fundamental physiology of this tissue. In addition to its important fundamental functions, the salivary glands of the black-faced leafhopper also express transcripts encoding proteins that might play a role in black-faced leafhopper-maize interactions. Based on similarity to salivary proteins in other leafhoppers and gene expression patterns, some of these transcripts likely encode important functions, such as antioxidant responses, detoxification, and calcium and cell wall degradation, which are unique features of Auchenorrhyncha. Finally, the presence of uncharacterized proteins within the predicted secretory peptides in leafhopper salivary glands suggest conservation and perhaps co-evolution within this group. Investigating the proteome of saliva and salivary glands will complement and enhance the transcriptomic evidence found in the black-faced leafhopper salivary gland. Nevertheless, this study provides a comprehensive foundation towards further understanding leafhopper–host interactions.

## Figures and Tables

**Table 1:** Summary of assembled salivary gland and reference transcriptomes of *Graminella nigrifrons*, black-faced leafhopper.

Assembly Parameters	Salivary Gland Transcriptome	Reference Transcriptome
Number of sequences	69,641	136,865
Length of the sequences	201–10,795	201–10,795
Number of bases	37,960,148	81,773,441
Number of transcripts with an Open Reading Frame	9102	22,283
N50 *	701	822
GC content	0.375	0.378

* N50 represents that 50 percent of transcripts have a length more than the respective number.

**Table 2:** Molecular function gene ontology terms (level 4) within the predicted secretome of black-faced leafhopper (*Graminella nigrifrons*). Secretome was predicted by identifying peptides with signal peptide (signalP v.5.0), absence of transmembrane helix (TMHMM v.2.0), and extracellular location (DeepLoc v.1.0). Blast descriptions were assigned as identified by B function against the non-redundant (nr) National Center for Biotechnology Information database.

Enriched Molecular Function Gene Ontology	Contig ID	Putative Blast Description
Hydrolase activity, acting on ester bonds	TRINITY_DN26981_c0_g1_i1	DNA repair protein RAD50-like
	TRINITY_DN29993_c0_g1_i1	Tyrosine-protein phosphatase 10D isoform X1
	TRINITY_DN16434_c1_g1_i1	Acid phosphatase-1
	TRINITY_DN24127_c0_g1_i1	Phosphatidyl glycerophosphatase and protein-tyrosine phosphatase 1
	TRINITY_DN26134_c0_g3_i1	Pancreatic lipase-related protein 2-like
Hydrolase activity, acting on glycosyl bonds	TRINITY_DN16637_c1_g1_i1	Cyclin-dependent kinase 8-like
	TRINITY_DN26139_c0_g7_i1	α-N-acetylgalactosaminidase
	TRINITY_DN24554_c0_g1_i1	Cellulose-binding protein
	TRINITY_DN21305_c0_g1_i2	Pre-mRNA branch site protein p14
Chitin binding	TRINITY_DN18138_c0_g1_i1	Chitin deacetylase 4
	TRINITY_DN17883_c0_g1_i1	Chitin deacetylase 3
	TRINITY_DN21305_c0_g1_i2	Pre-mRNA branch site protein p14
Peptidase activity	TRINITY_DN23314_c0_g1_i2	Cathepsin L
	TRINITY_DN25538_c0_g1_i1	Putative serine protease K12H4.7
	TRINITY_DN25934_c0_g1_i2	Cathepsin B
	TRINITY_DN19249_c1_g1_i1	Putative GPI-anchor transamidase
	TRINITY_DN24056_c0_g1_i1	Calpain-A-like isoform X6
	TRINITY_DN24709_c0_g1_i1	Cathepsin L
Cation binding	TRINITY_DN26981_c0_g1_i1	DNA repair protein RAD50-like
	TRINITY_DN20367_c0_g1_i1	Transferrin
	TRINITY_DN24056_c0_g1_i1	Calpain-A-like isoform X6

**Table 3:** Salivary gland-enriched transcripts of black-faced leafhopper (*Graminella nigrifrons*) adults fed on maize at P_adj_ < 0.05 and log_2_ fold change ≥ 5. Candidates were chosen based on the molecular function gene ontology categories with their identification in other hemipteran saliva.

Contig ID	NCBI Reference Accession	Putative Identification	Log_2_ Fold Change	P_adj_	SignalP Prediction	Transmembrane Domain Prediction
Cation binding						
TRINITY_DN29516_c0_g1_i1	G9M8X1.1	Calcium-binding protein SP84 (NcSP84)	10.94	5E-11	YES	NO
TRINITY_DN35919_c0_g1_i1	XP_031636025.1	Annexin B9 isoform X1	10.15	2E-20	YES	NO
TRINITY_DN25027_c0_g1_i1	XP_028676409.1	Calmodulin, putative	10.14	1E-29	YES	NO
TRINITY_DN22216_c0_g1_i1	BBH63273.1	Laccase-1 ^Ϯ^	10.01	4E-116	YES	NO
TRINITY_DN37325_c2_g6_i1	XP_016201745.1	Calmodulin-like protein 1	9.35	2E-10	YES	NO
TRINITY_DN37587_c0_g1_i4	XP_012151393.1	Prolyl 4-hydroxylase subunit α-2	9.23	2E-09	NO	-
TRINITY_DN39232_c0_g12_i2	XP_008555414.1	Angiotensin-converting enzyme-like isoform X1	8.83	7E-11	NO	NO
TRINITY_DN33957_c0_g1_i1	BAJ06131.1	Laccase 1 isoform S	8.57	3E-151	YES	NO
TRINITY_DN19685_c0_g1_i1	KFM77473.1	Calcium-binding protein E63-1 ^Ϯ^	8.38	2E-27	YES	NO
TRINITY_DN29669_c0_g1_i4	XP_022202851.1	EF-hand calcium-binding domain-containing protein 4A-like isoform X1	8.37	4E-05	NO	-
TRINITY_DN38312_c0_g3_i1	XP_026811683.1	Carbonic anhydrase 2-like	8.04	4E-14	YES	NO
TRINITY_DN29189_c0_g2_i1	XP_026272268.1	Prolyl 4-hydroxylase subunit α-1	7.91	6E-05	NO	-
TRINITY_DN25200_c0_g1_i1	XP_019167479.1	Calcium-binding allergen Ole e 8-like	7.39	5E-05	YES	NO
TRINITY_DN17761_c0_g1_i1	XP_033736401.1	Calmodulin-like protein 11	5.88	5E-04	YES	NO
TRINITY_DN13330_c0_g1_i1	XP_008484676.1	Uncharacterized protein K02A2.6-like, partial	5.33	1E-01	NO	NO
Hydrolase activity (Ester bonds)						
TRINITY_DN38399_c0_g1_i1	XP_022196409.1	Protein 5NUC-like	9.45	3E-19	YES	YES
TRINITY_DN35420_c0_g1_i4	XP_014273713.1	Alkaline ceramidase 3	8.34	4E-11	NO	-
TRINITY_DN27013_c0_g1_i1	XP_026275034.1	Pancreatic triacylglycerol lipase-like	12.23	5E-18	YES	NO
TRINITY_DN27304_c0_g1_i2	XP_022834705.1	Phospholipase A1-like	9.09	7E-14	YES	NO
TRINITY_DN36961_c0_g1_i2	XP_028657491.1	Deoxyribonuclease-2-α isoform X2	8.73	6E-11	NO	NO
TRINITY_DN39210_c0_g1_i1	XP_008195535.1	Inactive pancreatic lipase-related protein 1	8.33	3E-09	NO	NO
TRINITY_DN34634_c0_g2_i2	XP_021935199.1	Venom acid phosphatase Acph-1-like	8.30	7E-28	NO	NO
TRINITY_DN40024_c0_g1_i2	RZC33704.1	Venom carboxylesterase-6-like	8.17	3E-08	YES	NO
TRINITY_DN38228_c0_g1_i8	XP_024867821.1	Phospholipase A1 member A-like isoform X1	7.97	1E-07	YES	NO
Peptidase activity						
TRINITY_DN39232_c0_g8_i1	XP_029977452.1	Angiotensin-converting enzyme-like	11.24	7E-17	NO	NO
TRINITY_DN39315_c0_g1_i1	XP_030371519.1	Aminopeptidase N-like	9.80	8E-31	NO	-
TRINITY_DN39232_c0_g12_i2	XP_030751661.1	Angiotensin-converting enzyme-like isoform X1	8.83	7E-11	NO	NO
TRINITY_DN33549_c0_g5_i6	VVC42832.1	Peptidase S1, PA clan, Serine proteases, trypsin domain	8.46	8E-07	NO	NO
TRINITY_DN41406_c0_g4_i3	XP_031337350.1	Lysosomal aspartic protease-like	8.10	2E-07	YES	NO
TRINITY_DN38080_c1_g7_i1	XP_018910288.1	Zinc metalloproteinase nas-13-like	8.04	4E-06	NO	NO
TRINITY_DN41406_c0_g4_i1	VTJ90797.1	Hypothetical predicted protein	7.58	3E-05	YES	NO
TRINITY_DN32600_c0_g1_i2	XP_026203206.1	Pepsin A-like	6.70	5E-03	YES	NO
Others						
TRINITY_DN34735_c1_g5_i1	BAQ94509.1	NcSP19 ^Ϯ^	12.29	2E-115	YES	YES
TRINITY_DN37500_c0_g1_i1	BAQ94503.1	NcSP75 ^Ϯ^	9.31	4E-29	YES	NO
TRINITY_DN24271_c0_g1_i1	BAQ94508.1	NcSP22	10.77	2E-29	YES	NO
TRINITY_DN35988_c0_g2_i1	WP_103338510.1	Cellulase family glycosylhydrolase ^Ϯ^	8.90	2E-10	YES	NO
TRINITY_DN34815_c0_g1_i1	RZF49131.1	Unknown protein	10.48	4E-12	NO	-
TRINITY_DN27409_c0_g3_i1	XP_022196219.1	Uncharacterized protein LOC111053608	10.29	3E-10	NO	-

^Ϯ^ Expression of these transcripts was validated in the adults fed on maize plants relative to starved by real time quantitative PCR.

## Supplementary Materials

The following are available online at https://www.mdpi.com/2073-4425/11/11/1289/s1: Supplementary File 1: Table S1: Raw and trimmed reads obtained from deep sequencing of salivary glands (SG) and carcass (CC) of the black-faced leafhopper fed on maize. Table S2: Benchmarking Universal Single-Copy Orthologs (BUSCO, v.3.1.0) and TransRate (v.1.0.3) output for the reference transcriptome. Table S3: Extracellular space and extracellular region proteins in the salivary gland of black-faced leafhopper. Table S4: Primers used for quantifying the expression of candidate transcripts in maize-fed and starved black-faced leafhopper adults. Supplementary File 2: Table S5: Putative secretory proteins in the salivary gland transcriptome of *Graminella nigrifrons* identified by the presence of signal peptide (SignalPv.5.0), no transmembrane domain (THMM v.2.0), and extracellular localization (DeepLoc v.1.0). Peptides are annotated in Blast2GO using Blastp algorithm against a non-redundant database. Table S6: Differentially expressed transcripts identified by DESeq2 (v.1.15.46) and annotated by BLASTx. Salivary gland (Gn_SG) tissue was treated as treatment and carcass tissue (Gn_CC) as control. Tissues were collected from male and female black-faced leafhopper adults feeding on maize plants.
