# Supplementary material for: Transcriptome Analysis Reveals Functional Diversity in Salivary Glands of Plant Virus Vector, Graminella nigrifrons
Source: Genes (Basel). 2020 Oct 29;11(11):1289. doi: 10.3390/genes11111289 (PMC7716219; doi:10.3390/genes11111289)
Supplement: Supplementary file 1 [file genes-11-01289-s001.zip › Rajarapu_etal_Supplementary files/Rajarapu et al_Supplementary file 1.docx]

*For submission to Genes*

**Transcriptome analysis reveals functional diversity in salivary glands of a plant virus vector**

Swapna Priya Rajarapu^1,4^, Raman Bansal^2,4^, Priyanka Mittapelly^3,4^, Andrew Michel^4,5^

^1^Current address: Department of Entomology and Plant Pathology, North Carolina State University, Raleigh, North Carolina, 27606

^2^Current address: USDA, Agricultural Research Service, San Joaquin Valley Agricultural Sciences Center, 9611 South Riverbend Avenue, Parlier CA 93648-9757

^3^Current address: USDA-APHIS PPQ, 5936 Ford Court, Suite 200, Brighton, Michigan, 48116

^4^Department of Entomology, ^5^The Center for Applied Plant Sciences, OARDC, The Ohio State University, Wooster Ohio, 44691

Corresponding author:

Swapna Priya Rajarapu

1680 Madison Ave

Wooster, OH 44691

Phone: 330-263-3686

Email: rajarapu.1@osu.edu

rajarapuswapna@gmail.com

Current address:

840 Main Campus Dr

Partners II, Ste 1400

Raleigh, NC 27511

Supplementary Tables and Figures:

**Table S1:** Raw and trimmed reads obtained from deep sequencing of salivary glands (SG) and carcass (CC) of the black-faced leafhopper fed on maize.

|  |  | **RAW READS** | |  | **TRIMMED READS** | |  |
| --- | --- | --- | --- | --- | --- | --- | --- |
| Treatment | Rep | Paired end file | # of reads | % GC | # of reads | % GC | % Retained reads |
| CC | 1 | S1 | 6,258,442 | 38 | 5,520,166 | 37 | 88.2 |
|  |  | S2 | 6,258,442 | 37 | 5,520,166 | 37 |  |
|  | 2 | S1 | 7,960,404 | 39 | 6,990,787 | 38 | 87.8 |
|  |  | S2 | 7,960,404 | 38 | 6,990,787 | 38 |  |
|  | 3 | S1 | 5,838,126 | 39 | 5,155,080 | 38 | 88.3 |
|  |  | S2 | 5,838,126 | 38 | 5,155,080 | 38 |  |
| SG | 1 | S1 | 9,547,975 | 46 | 8,239,344 | 46 | 86.3 |
|  |  | S2 | 9,547,975 | 46 | 8,239,344 | 46 |  |
|  | 2 | S1 | 8,276,214 | 49 | 7,068,699 | 48 | 85.4 |
|  |  | S2 | 8,276,214 | 49 | 7,068,699 | 48 |  |
|  | 3 | S1 | 8,609,536 | 47 | 7,383,994 | 47 | 85.8 |
|  |  | S2 | 8,609,536 | 47 | 7,383,994 | 47 |  |

**Table S2:** Benchmarking Universal Single-Copy Orthologs (BUSCO.v.3.1.0) and TransRate (v.1.0.3) output for individually assembled and merged transcriptomes (merged assembly) and combined transcriptome (combined assembly).

| **BUSCO** | **Combined assembly** |
| --- | --- |
| Complete BUSCOs | 1332  (80.4%) |
| Complete and single-copy BUSCOs | 1087  (65.6%) |
| Complete and duplicated BUSCOs | 245  (14.8%) |
| Fragmented BUSCOs | 231  (13.9%) |
| Missing BUSCOs | 95  (5.7%) |
| Total BUSCO groups searched | 1658 |
| **TransRate** |  |
| Number of sequences | 103,463 |
| Length of the sequences | 201-10795 |
| Number of bases | 65,646,610 |
| Number of transcripts with an ORF | 17,552 |
| N50* | 920 |
| GC content | 0.378 |

**Table S3:** Extracellular space and extracellular region proteins in the salivary gland of black-faced leafhopper

| Extracellular space | |
| --- | --- |
| TRINITY_DN1002_c0_g1_i1 | carboxypeptidase E |
| TRINITY_DN11348_c0_g1_i1 | CD109 antigen-like isoform X1 |
| TRINITY_DN14424_c0_g1_i1 | mesencephalic astrocyte-derived neurotrophic factor homolog |
| TRINITY_DN16037_c0_g1_i1 | cathepsin B-like |
| TRINITY_DN19625_c0_g1_i1 | cathepsin L |
| TRINITY_DN19904_c0_g1_i1 | carboxypeptidase E-like |
| TRINITY_DN20348_c0_g1_i1 | dnaJ homolog subfamily B member 11 |
| TRINITY_DN20367_c0_g1_i1 | transferrin |
| TRINITY_DN20785_c0_g1_i1 | prothoracicostatic peptide |
| TRINITY_DN20785_c0_g1_i2 | prothoracicostatic peptide |
| TRINITY_DN21070_c0_g1_i3 | S-formylglutathione hydrolase |
| TRINITY_DN21521_c0_g1_i1 | superoxide dismutase [Cu-Zn] |
| TRINITY_DN21870_c0_g1_i1 | SPARC |
| TRINITY_DN21870_c0_g1_i3 | SPARC |
| TRINITY_DN22915_c0_g3_i1 | peptidyl-prolyl cis-trans isomerase 6 |
| TRINITY_DN24784_c0_g1_i1 | FMRFamide-related peptides |
| TRINITY_DN25042_c0_g2_i1 | transferrin |
| TRINITY_DN25851_c0_g1_i1 | palmitoyl-protein thioesterase 1 |
| TRINITY_DN25934_c0_g1_i2 | cathepsin B |
| TRINITY_DN26155_c0_g1_i1 | endoplasmin |
| TRINITY_DN40095_c0_g1_i1 | calreticulin |
| TRINITY_DN42503_c0_g1_i1 | melanotransferrin |
| TRINITY_DN44088_c0_g1_i1 | melanotransferrin |
| TRINITY_DN651_c0_g1_i1 | serpin B3-like isoform X1 |
| Extracellular region | |
| TRINITY_DN11046_c0_g1_i1 | Alpha-2-macroglobulin receptor-associated protein |
| TRINITY_DN18384_c0_g1_i2 | ankyrin-2-like isoform X3 |
| TRINITY_DN18384_c0_g1_i1 | Ankyrin-2-like Protein |
| TRINITY_DN3243_c0_g1_i1 | bone morphogenetic protein 3 |
| TRINITY_DN22985_c0_g1_i1 | cartilage oligomeric matrix protein |
| TRINITY_DN11222_c0_g1_i1 | chitin deacetylase 1 |
| TRINITY_DN11222_c1_g1_i1 | chitin deacetylase 1 |
| TRINITY_DN19263_c0_g1_i1 | chitin deacetylase 2 |
| TRINITY_DN17883_c0_g1_i1 | chitin deacetylase 3 |
| TRINITY_DN18138_c0_g1_i1 | chitin deacetylase 4 |
| TRINITY_DN16320_c0_g1_i1 | chitin deacetylase-like 5 |
| TRINITY_DN16320_c0_g2_i1 | stress response protein nst1 isoform X1 |
| TRINITY_DN21305_c0_g1_i2 | Pre-mRNA branch site protein p14 |
| TRINITY_DN1801_c0_g1_i1 | probable chitinase 3 |
| TRINITY_DN3130_c0_g1_i1 | Collagen alpha-1(IV) chain |
| TRINITY_DN15421_c0_g1_i1 | DNA-binding protein RFXANK, putative |
| TRINITY_DN21081_c0_g1_i1 | glutaminyl-peptide cyclotransferase |
| TRINITY_DN18242_c0_g1_i1 | orcokinin peptides type A-like |
| TRINITY_DN18242_c0_g1_i2 | orcokinin peptides type A-like |
| TRINITY_DN2980_c0_g1_i1 | pancreatic lipase-related protein 2-like |
| TRINITY_DN11187_c0_g1_i1 | pancreatic triacylglycerol lipase-like isoform X2 |
| TRINITY_DN1710_c0_g2_i1 | papilin isoform X1 |
| TRINITY_DN32409_c0_g1_i1 | protein slit |
| TRINITY_DN14097_c0_g1_i1 | Splicing factor 3A subunit 3 |
| TRINITY_DN29383_c0_g1_i1 | Vesicle-fusing ATPase 1 |

**Table S4:** Primers used for quantifying the expression of transcripts that could contribute to host feeding in maize fed and starved *Graminella nigrifrons*, black-faced leafhopper adults

| **Name** |  | **Primer sequence (5´ to 3´)** | **Tm (°C)** | **Product length (bp)** | **E** |
| --- | --- | --- | --- | --- | --- |
| *GnP19* | Forward | CAGTGACCGCCAAGTCCTAT | 56.8 | 105 | 1.96 |
|  | Reverse | TACTTGTTGCCAGCAGCATC | 55.8 |  |  |
| *GnP75* | Forward | CGATACGGCTAAAAGCAAGC | 54.3 | 82 | 2.19 |
|  | Reverse | TTTTGATGCCGCTAATTTCC | 51.6 |  |  |
| *GnLac1* | Forward | GCTACCAACAAAACCCGAGA | 55.2 | 87 | 1.94 |
|  | Reverse | GTCCACAGGGTTGAAGTCGT | 57.2 |  |  |
| *GnE63* | Forward | AATGACGCTTTGCTCAAGGT | 55.2 | 146 | 1.87 |
|  | Reverse | TCCATGAGCTCGTCAATCTG | 54.5 |  |  |
| *GnGHF5* | Forward | CGTGTCGTGGCAAATACAAC | 54.6 | 100 | 1.99 |
|  | Reverse | GACGTGGAACAACTGGGACT | 57.2 |  |  |
| *GnRPS13* | Forward | TCCCAGTCTGCTCTTCCCTA | 57.1 | 94 | 1.83 |
|  | Reverse | CACCGTGAGAGTCCCTCAAT | 56.5 |  |  |
